# Underestimating the Toxicological Challenges Associated with the Use of Herbal Medicinal Products in Developing Countries

**DOI:** 10.1155/2013/804086

**Published:** 2013-09-19

**Authors:** Vidushi S. Neergheen-Bhujun

**Affiliations:** Department of Health Sciences, Faculty of Science and ANDI Centre of Excellence for Biomedical and Biomaterials Research, University of Mauritius, Reduit, Mauritius

## Abstract

Various reports suggest a high contemporaneous prevalence of herb-drug use in both developed and developing countries. The World Health Organisation indicates that 80% of the Asian and African populations rely on traditional medicine as the primary method for their health care needs. Since time immemorial and despite the beneficial and traditional roles of herbs in different communities, the toxicity and herb-drug interactions that emanate from this practice have led to severe adverse effects and fatalities. As a result of the perception that herbal medicinal products have low risk, consumers usually disregard any association between their use and any adverse reactions hence leading to underreporting of adverse reactions. This is particularly common in developing countries and has led to a paucity of scientific data regarding the toxicity and interactions of locally used traditional herbal medicine. Other factors like general lack of compositional and toxicological information of herbs and poor quality of adverse reaction case reports present hurdles which are highly underestimated by the population in the developing world. This review paper addresses these toxicological challenges and calls for natural health product regulations as well as for protocols and guidance documents on safety and toxicity testing of herbal medicinal products.

## 1. Introduction 

With the burst in the use of herbal medicinal products (HMPs) globally, either for primary treatment or as complementary and alternative medicine, safety and efficacy of herbal medicine have become a public health concern. Till date, it has been difficult to make reliable estimates of ill health caused by herbal products particularly because (i) the perception that “natural” equates to “safe” and therefore herbal medicine users would not realise that a herbal remedy may be responsible for adverse symptoms they have experienced; (ii) the lack of communication between patients and medical practitioners regarding the use of herbal medicine; (iii) the presence of low-grade herbal products on the market, and (iv) the supply of counterfeit products. 

The World Health Organisation (WHO) estimates that 80% of Asian and African populations rely on traditional medicine as the primary method for health care needs. The scenario in developed countries is very similar with 70% to 80% of the population using some form of complementary and alternative medicine [1]. Commonly used single herbs and polyherbal formulations in developing nations are described in Tables 1 and 2. Whilst conventional medical science has powerful methodologies for proving efficacy, ensuring quality, standardising good manufacturing practices, testing for safety, and conducting postmarketing surveillance for adverse effects, similar guidelines are lacking for herbal products; the same has not been extended to traditional herbal medicines despite these having been embraced by different cultures and regions. It must, however, be acknowledged that a number of protocols documents on safety and toxicity testing of HMPs have been put forward by the Union of Pure and Applied Chemistry [2], the European Medicines Agency (EMEA) [3], and the European Food Safety Authority [4] though they are not followed by all countries. In addition, with the rising demand of medicinal plants from developed countries, international trade particularly via the Internet has soared. However international trade in medicinal plants is not well regulated, with limited data available on the product identity, on the true demand and supply, and on the price of the unprocessed raw materials and the processed HMPs [5]. Although there exists the WHO certification scheme to regulate the quality of HMPs in international commerce, noncompliance with this certification further accentuates the problems of adverse reactions [6].

Adverse health effects associated with herbal products exist since time immemorial though and could be attributed to both the inherent toxic effects of herbal medicine and toxicities induced by adulterants/contaminants. The low incidence of adverse reports associated with HMPs in developing countries may be explained by the fact that consumers generally regard them as safe and therefore believe their symptoms are not attributable to the use of those products. In addition, the reluctance to indicate the concomitant use of HMPs to health care professionals, the impression that the adverse effects are known, forgetfulness, unwillingness to report based on suspicion alone, pressures of clinical practice, and uncertainty about the reporting process are key factors which if addressed can certainly help in risk mitigation. Whilst evidence-based studies indicating the efficacy of herbal drugs are still being unveiled, increasing evidence, regarding side effects of herbal medicine, has highlighted the demand for and consequently the necessity of toxicological studies for herbal products. 

With the increased discussion on safety assessment of herbs, toxicology constitutes an essential role in the development of herbal medicines and advancements of analytical techniques and molecular technology, in particular the “-omic-” technology comprising transcriptomics, proteomics, and metabonomics, can make a significant contribution to the predictive and preclinical toxicology assessment of herbal medicine [28, 29].

Apart from the toxicity of the intended herb(s) itself, the lack of a stringent and harmonised quality control and effective monitoring system imposed on herbal medications may lead to contamination or adulteration that could prove harmful to humans. Possible contaminants/adulterants such as heavy metals, pesticides, toxic herbs, and conventional drugs are commonly encountered toxicological concerns in herbal preparations. Nevertheless, strategies in devising suitable toxicological examination protocols to deal with the wide panoply of components in herbal drugs stand as a challenge to toxicologists.

Heavy metal contaminants like cadmium (Cd), arsenic (As), and lead (Pb) can be a risk factor in contributing to the toxicity of these herbal products [30]. The WHO maximum permissible limits of As, Cd, and Pb are 1.0, 0.3, and 10 ppm, respectively [31], with agricultural practices and industrial emissions being accounted as indirect contributors [32, 33]. Hence, a recent study by Affum et al. [34] showed the level of arsenic in ready-to-use aqueous-based antimalaria herbal medicine in Ghana to be above the WHO permissible levels. Plant material may also be contaminated with pesticides and subsequent poor manufacturing practices may lead to contamination with bacteria, fungi, and other microorganisms. In addition, an increasing number of herbal supplements have been found to be adulterated with active pharmaceutical products [35]. 

Case reports have played a crucial role in alerting the scientific community on the adverse effects of therapeutic interventions [36, 37]. This is particularly true for herbal medicines, many of which have a long traditional use but without ever having been submitted to formal tests of safety compared to conventional medicines. Moreover, despite their importance, case reports are often of poor quality, a fact that can seriously limit their value [38]. Hence, many of the case reports in the developing countries are not properly recorded, limiting appropriate conclusion thereby contributing to increasing adverse clinical effects of herbal remedies.

## 2. Evidence-Based Herbal Drug Toxicity

Toxicological problems associated with the use of herbal medicines are complex but have been regularly associated with serious adverse fatalities ranging from cardiovascular problems to psychiatric to neurological effects to liver toxicity or malfunction to hematologic and renal toxicity [39–41]. The diagnoses of herbal toxicity are usually made after consideration of the temporal relationship between exposure to the herb and the occurrence of the adverse event and by excluding other causes. However, causality assessment using appropriate tools for ascertaining herbal toxicity in a number of cases has failed to show any causal effects or has indicated only weak causal relationship [42, 43]. For instance, the report of toxic liver injury due to consumption of the herb Greater Celandine (*Chelidonium majus* L.) in patients from various European countries has been a matter of concern. Teschke et al. [43] provided evidence of the existence of Greater Celandine hepatotoxicity as a distinct form of herb-induced liver injury in 22 spontaneous cases in Germany, but due to poor data quality the causal association between the herb usage and liver injury was less strong than hitherto assumed. 

Moreover, although weak or no causality of herbal toxicity could be shown in many instances, a number of studies still have reported the potentially toxic nature of herbal drugs. In 1992, in Belgium, consumers of a herbal weight-loss preparation containing *Aristolochia *spp. exhibited severe renal disease manifested by interstitial fibrosis, which rapidly progressed to renal failure [44]. Moreover, *Aristolochia *spp. also used as an aphrodisiac, as an anticonvulsant, as an immune stimulant, and to treat arthritis, gout, rheumatism, eczema, wound treatment, allergic gastrointestinal colic, and gallbladder colic have been subsequently reported to impair renal function due to the presence of aristolochic acid [45, 46]. In 1995, in Southern Taiwan and Japan, the use of *Sauropus androgynus* in weight control was associated with an outbreak of *Sauropus androgynus*-related obstructive lung disease [47]. Jones and Lawson [48] reported that the use of blue cohosh herbal medication was associated with profound neonatal congestive heart failure. The effect could be attributed to the presence of vasoactive glycosides and to an alkaloid known to produce toxic effects on the myocardium. Furthermore, blue cohosh has sympathomimetic and direct cardiotoxic effects, which can cause coronary vasoconstriction and decrease oxygen flow to the heart, thereby leading to morbidity and mortality in the foetus or in newborn infant exposed via maternal ingestion [48]. Similarly, cardiac glycosides have been linked with hyperkalaemia, a side effect observed in a patient taking a long list of herbal medicinal drugs [49].

Similarly, in 2005, clinical problems arising from the use of herbal medicines were reported in Hong Kong and *Aristolochia* species was found responsible for acute renal failure (*n* = 1), with aconite roots causing aconitine poisoning (*n* = 2), the *Datura* species causing anticholinergic poisoning (*n* = 1), and “yulan” (*Stephania sinica*) causing tetrahydropalmatine poisoning (*n* = 3) [50]. Likewise, in 2007, a systematic survey in Swiss hospitals indicated 10 cases implicating Herbalife, a herbal product sold for promoting “wellness” and weight reduction, as a possible cause of potentially severe hepatotoxicity. However it should be noted that the causality assessment in these cases was conducted by the WHO global introspection method, which is not liver-specific and therefore not a reliable tool to ascertain causality in presumed hepatotoxicity cases. Causality assessment of hepatotoxicity cases requires the Council for International Organisations of Medical Sciences (CIOMS) methods. Thus, for the 10 cases, a number of parameters for a valid causality assessment were not reported and there was also a case of hepatitis E explaining the presence of liver disease [51]. 

The diagnosis of herbal-drug-induced hepatotoxicity continues to be a challenge despite the availability of causality assessment tests. There are only limited number of clinical studies with HMPs reported in the literature despite the fact that they have been used for centuries. Thus postmarket pharmacovigilance providing interesting source of safety information and causality assessment indicates a link between an observed adverse event to a suspected HMP [52]. Although there is no universally accepted method for causality assessment, the existing methods rely on algorithmic, probability-based, and expert analyses as well as on the quality of adverse reactions reports. Unless causality is established, narrative reports remain less convincing of any herbal adverse reactions.

Cases with severe intoxications in humans have also been reported after consumption of essential oil rich in thujone. Thujone or thujone-containing products have been reported to cause central nervous system disturbances which can lead to convulsions and ultimately to unconsciousness and death [53]. Similarly, licorice (*Glycyrrhiza glabra*) commonly used for inflammation of the upper respiratory tract and gastric and duodenal ulcers has been reported to cause suppression of the renin-aldosterone system, resulting in sodium and water retention, hypokalemia, hypertension, cardiac arrhythmias, and myopathy in cases of prolonged use [54, 55]. Also, 10 deaths and 13 permanent disabilities from Ephedra-containing herbal drugs were reported to the FDA and the effects with ascribed to its sympathomimetic effects [41, 56]. Overall, it is seen that while, on one hand, the toxic effects of herbs have been widely reported in developed countries, the same has not received an equivalent depth of scrutiny in developing countries.

The toxicity of herbs may also result from the generation of reactive intermediates through metabolic activation of herbal constituents via phases I and II reactions within the human body. The resultant reactive intermediates can bind covalently to DNA and proteins, leading to organ toxicity, mutagenicity, and even carcinogenicity. For instance, aristolochic acids in *Aristolochia* spp. used in a number of Chinese traditional medicine undergo reduction of the nitro group by hepatic CYP1A1/2 or peroxidases in extrahepatic tissues generating highly reactive cyclic nitrenium ions. The latter can react with DNA to form promutagenic DNA adducts such as 7-(deoxyadenosin-N6-yl) aristolactam I and 7-(deoxyguanosin-N2-yl) aristolactam I as well as protein, resulting in activation of H-ras and myc oncogenes and gene mutation in renal cells and finally carcinogenesis of the kidneys [57, 58]. In vitro studies have also indicated the role of herbal reactive intermediates in irreversibly inhibiting various cytochrome enzymes (CYPs). However, the discrepancy of effects between in vitro, animal, and human studies reflects the significance of herbal dosing in the modulation of CYPs [58].

Other factors compromising safety may result from herbs harvested from polluted sites or poor farming practices, medicinal plant products contaminated with pesticides and microbial contaminants, heavy metals, toxic substances, and adulterants which can be toxic to the consumer.

## 3. Evidence-Based Drug Herbal Interactions

**Figure 1:**
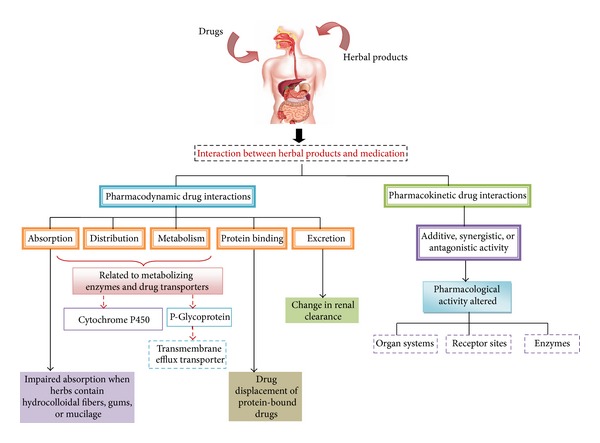
Generalised mechanistic insight into herb-drug interactions.

The contemporary use of herbal medicine is widespread but the challenge that society faces with its use is whether a patient will divulge to his or her medical practitioner the concurrent use of herbal products with conventional pharmacotherapy. A number of observational studies have shown the negative or conflicting interactions of drugs and herb though theoretically herb-drug interactions could also be positive or neutral [59]. Herbal drugs usually contain a multitude of pharmacologically active ingredients, a fact that greatly increases the possibilities of interactions. In many instances, the likelihood of herb-drug interactions could be higher than drug-drug interactions. Interactions between herbs and drugs may increase or decrease the pharmacological or toxicological effects via the pharmacokinetic herb-drug interactions caused by one medicine interfering with the elimination, metabolism, or absorption of another medicine and the pharmacodynamic herb-drug interactions caused by two different medicines working in the same or opposite directions and ultimately affecting the dose response and any mechanisms of therapeutic or toxic effects (Figure 1) [35]. For example, St-John's Wort, a well-known cytochrome P450 inducer, may affect systemic bioavailability of many conventional drugs [60].

Common examples of herb-drug interactions have been found when cardiovascular medications with a narrow therapeutic index, such as digoxin and warfarin, are coadministered with herbs. For instance, concomitant use of warfarin with St. John's Wort decreases prothrombin time, which may result in reduced anticoagulant effect and need for increased warfarin dose [61]. Other examples of interactions include St. John's Wort with cyclosporine [62] and grapefruit juice with felodipine and lovastatin [63].

The current evidence that herbal medicine may interact with conventional drugs is mainly based on case reports of patients, case series, and a limited number of clinical studies. Drug-herbal interactions are difficult to evaluate because of the lack of compositional reliability of the herbal products though a number of interactions, amongst which are drug-metabolizing enzymes and drug transporter systems, as well as pharmacodynamic interactions can be involved. However since the pharmacokinetic and pharmacodynamic characteristics of most herbal medicine or supplements are not completely recognized, potential interactions cannot be predicted.

In a clinical study involving 313 patients, Jeong et al. [64] concluded that herbal drugs used alone were relatively safe, but the risk for adverse reactions may increase when herbal and conventional drugs are taken concurrently. The results indicated a 2.3% incidence of liver injury in the combined group of Korean patients. Concomitantly, Zhu et al. [65] reported that rutaecarpine, an alkaloid found in *Evodia rutaecarpa* traditionally used in combination with Chinese traditional medicine, had profound effects on the hepatic drug processing enzyme gene expression, CYP enzyme genes and UDP-glucuronosyltransferase, and increased the expression of hepatic uptake and efflux transporters. The authors speculated that all these effects could play an integrated role in rutaecarpine-increased metabolism and elimination of caffeine [66], theophylline [67], and acetaminophen [68].

From evidence-based herb-drug interaction in cancer chemotherapy, Cheng et al. [59] concluded that most of the available information, both positive and negative, came from basic in vitro experiments or trials testing the use of a single herb along with chemotherapy drug when in practice most of the herbs are used in mixtures. The latter argued that “it is not reasonable to discard the potential usefulness of the traditional wisdom of herbal medicine, which has the backing of thousands of years of clinical experience.” However, this statement should be taken with some level of skepticism particularly since clinically relevant pharmacokinetic interactions between anticancer drugs and CAM have already been reported between the frequently used St. John's Wort and the anticancer drugs irinotecan [69] and imatinib [70]. This justifies the need for clinical studies for confirmation and assessment of the clinical relevance of CAM-drug interactions obtained in vitro and emphasized recently by Goey et al. [71].

## 4. The Way Forward For Developing Countries

Due to the wide use and easy availability of herbal medicines, herbal toxicity and herb-drug interactions have become an issue of global concern. While HMPs provide opportunities for complementing the armoury of existing drugs, for their purportedly preventive and therapeutic purposes, the major problem with the use of herbal-based treatments is the lack of definite and complete information about the composition of extracts, resulting mainly from the trade secrecy of a number of herbal practitioners. Despite the fundamental role that traditional medical practitioners play in the provision of health services in developing countries, trade secrecy has been observed and is currently encroaching on the proper assessment of local herbal drugs. The lack of Good Manufacturing Practices, Good Agricultural Practices, Good Laboratory Practices, and metabolic, pharmacokinetic, and toxicologic characteristics further characterized the dangers of HMPs in developing countries.

Hence, in many developing countries involved with the active production and use of HMPs, regulations distinct from the one for food and drugs are warranted. The existence of such regulation can emphasise a premarket system, which provides market authorisation for each HMP based on evidence that the product is safe under the recommended conditions of use without a prescription, effective for the proposed claims, and of high quality, and is mandatory. In addition, a licence issued on the basis of evidence of compliance with Good Manufacturing Practices to importers, manufacturers, packagers, and labellers of HMPs will certainly aid in reducing health-related risks. Such an effort should be in concert with WHO International Regulatory Cooperation for Herbal Medicines (IRCH), established in 2005 to protect and promote public health and safety [72].

There is also a strong need for scientific evidence of the pharmacological qualities and safety of herb-derived remedies employed for centuries as traditional medicines in these countries. This is particularly because sound knowledge of the mechanisms of actions and interactions is essential for a clinical risk assessment. In addition, the design of an appropriate postmarket pharmacovigilance system will help address the fatalities of herbal drugs. Surveillance for HMP-related adverse responses should consist mainly of prompting voluntary reporting from consumers and health care practitioners. It should be noted that even if the interactions between medicinal herbal products and drugs may be clinically insignificant, susceptibility to them may be enhanced by a wide variety of patient-related factors, such as the presence of multiple diseases, other pharmacotherapies, or genetic predisposition.

At the same time, it would be helpful to set up a national and/or regional accessible database to document the use of herbal medicines. Concurrently, efforts should be made to educate both healthcare professionals and patients about the use of herbal medicine. It should also be emphasised that besides its role in primary healthcare, traditional medicines have been and continue to be a strategic option in drug discovery [73].

The previously mentioned long-term strategies could certainly help to address the toxicological concern of herbal drugs in developing countries. But more immediate actions are also warranted and should be geared towards (1) greater communication between patients and physicians about the use of herbal medicine, (2) health care professional should be aware of herb-drug interactions and encourage patients to discuss herbal medicine use, (3) physicians should question patients about their use of herbal medicines, and (4) surveillance of HMP-related adverse responses should be monitored. In the meantime, preclinical and clinical studies should be conducted to ascertain herb-drug interactions and government regulatory authority should put more efforts into natural health product regulations.

## Figures and Tables

**Table 1:** List of selected medicinal plants used as single herbs in developing countries.

Country	Family	Scientific name of plant	Vernacular name	Parts used	Local medicinal uses	Reported literature
Mauritius Island	Rutaceae	*Aegle marmelos *	Bael	Fruit Leaf	Gastrointestinal disorder Diabetes	[7–10]
	Erythroxylaceae	*Erythroxylum laurifolium *	Bois de ronde		Diuretic, used against renal stores	[11, 12]
	Ebenaceae	*Diospyros neraudii, Diospyros revaughanii, Diospyros tesselleria, Diospyros melanida *	Ebène	Stem bark	Antibacterial, antifungal, antiviral, anthelminthic, antiprotozoal, and antimalarial	[11–13]

Reunion Island	Aphloiaceae	*Aphloia theiformis *	Change ecorce, Goyave marron	Leaf	Treat fever and antimalarial properties	[14]
	Asteraceae	*Eupatorium triplinerve *	Ayapana	Aerial parts		

Madagascar Island	Acanthaceae	*Justicia gendarussa *	Ayapana marron	Aerial parts	Antimalarial properties	[15]
	Buddlejaceae	*Nuxia sp. *	Vlier	Leaf, bark		
	Asteraceae	*Psiadia sp. *	Arina/Iary	Aerial parts		

South Africa	Podocarpaceae	*Podorcarpus sp. *	Fern pine	Leaf Stem	Fevers, asthma, cough, cholera, arthritis, rheumatism, painful joints	[16]
	Apocynaceae	*Carissa edulis *	Conkerberry	Root	Diarrhoea	[17]

East Africa	Rutaceae	*Toddalia asiatica *	Nyalwet-kwach/Kaule/Mdaka komba	Root, bark, leaf, fruit	Malaria, cough, chest pain, sore throat	[18]
	Annonaceae	*Uvaria scheffleri *	Mguma	Root Leaf	Antimalarial activities	[19]

North Africa	Apiaceae	*Carum carvi *	El-qarwiya (Caraway)	Seed	Diabetes and hypertension	[20]
	Compositae	*Artemisia herba-alba *	Chih (White mugwort)	Leaf, root		

West Africa	Annonaceae	*Annickia chlorantha *	Yellow Moambe	Stem Bark	Aches, wounds, boils, vomiting, fever, chills, sore, spleen in children, and hepatitis	[21]
		*Anonidium mannii *	Eborne Afan	Stem Bark	Measles, diarrhea, enlarged spleen, fever, gonorrhoea, production of breast-milk	

Northeast India	Acanthaceae	*Justicia adhatoda *	Nongmangkha angouba	Leaf Fruit	Asthma	[22]
	Acoraceae	*Acorus calamus *	Ok hidak	Root Leaf	Haemorrhoids	
	Meliaceae	*Aphanamixis polystachya *	Heirangkhoi	Leaf	Asthma	

South India	Euphorbiaceae	*Acalypha indica *	Kuppaimeni	Leaf	Bronchitis	[23]
	Amaranthaceae	*Aerva lanata *	Sirukanpeelai	Root	Diabetes	
	Acanthaceae	*Asystasia gangetica *	Medday keerai	Whole plant	Rheumatism	

China	Rosaceae	*Agrimonia pilosa *	Xian he cao	Whole plant	Anti-inflammatory, against worms	[24]
	Asteraceae	*Erigeron breviscapus *	Dengzhanxixin	Whole plant	Inflammation, high blood pressure, and headache	
	Geraniaceae	*Geranium strictipes *	Geshanxiao	Root	Gastrointestinal disorders	

**Table 2:** Selected polyherbal formulation used in traditional medicine in developing nations.

Country	Polyherbal formulation	Herbal composition	Vernacular name	Parts used	Medicinal purpose	Reported literature
India	Polyherbal hepatoprotective formulation (PHF)	*Emblica officinalis Terminalia chebula Terminalia bellirica Picrorhiza kurroa * * Tinospora cordifolia * *Swertia chirata, Azadirachta indica, * *Adhatoda vasica *	Aamla Haritaki Vibhitaka Kutki Guduchi Felworts Neem Adusa	Fruits Fruits Fruits Stem Rhizomes Entire herb Bark Stem bark	Hepatic disease	[25]

China	Wu-Zi-Yan-Zong	*Cuscuta chinensis* *Lycium barbarum* *Rubus chingii* *Schisandra chinensis* *Plantago asiatica* *Epimedium brevicornum *	Strangleweed Chinese wolfberry Raspberries Pinyin Chinese plantain Yin Yang huo	Fruit Fruit Fruit Fruit Fruit Herb	Neuroinflammatory disease	[26]

Pakistan	PHOE (Polyherbal oil extract)	*Linum usitatissimum Trachyspermum ammi * *Myristica fragrans * *Syzygium aromaticum * *Colchicum luteum * *Celastrus paniculata * *Pinus roxburghii *	Alsi, Tuke Katan Ajwain Desi Jawatri Long Suranjan Talkh Mal Kangni Behroza	Seeds Seeds Seeds Flower buds Roots or tuber Seeds Oleo-resin	Antinociceptive and anti-inflammatory	[27]
